# Dominant inheritance and intra–familial variations in the association of Sturge–Weber and Klippel–Trenaunay–Weber syndromes

**DOI:** 10.4103/0971-6866.64943

**Published:** 2010

**Authors:** José Maria Pereira de Godoy, Agnes Cristina Fett-Conte

**Affiliations:** Adjunct Professor of the Cardiology and Cardiovascular Surgery Department and Professor of the Post Graduation course of Medical School of São Jose do Rio Preto-SP-FAMERP and CNPq Researcher-Brazil; 1 Molecular Biology Department Medical School, FAMERP, Brazil

**Keywords:** Genealogical study, Klippel–Trenaunay–Weber syndrome, Sturge–Weber syndrome

## Abstract

This case report shows a genealogical study where a woman has limb hypertrophy and her son has an association of Sturge–Weber syndrome with Klippel–Trenaunay–Weber syndrome. The Sturge–Weber and Klippel–Trenaunay–Weber syndromes appear to be different manifestations of the same affliction. Familial aggregation exists and transmission may be almost imperceptible between generations. Identification of minor manifestations may prove to be a valuable contribution to genetic counseling of families and the prevention of new cases.

## Introduction

The clinical manifestation of Klippel–Trenaunay–Weber Syndrome (KTW-OMIM 149000) includes irregular and asymmetrical capillary and cavernous hemangiomas on the trunk or limbs, arteriovenous fistulae, lymphedema, varicosities, asymmetrical hypertrophy and visceromegaly.[1,2] Sturge–Weber Syndrome (SW-OMIM 185300), also called encephalofacial or encephalotrigeminal angiomatosis, is characterized by purple-colored flat cutaneous cranial (face) hemangiomas most commonly along the trigeminal nerve, glaucoma and vascular lesions in the ipsilateral brain and meninges. Affected individuals can also present with meningeal angiomas, macrocephaly, buphthalmos, seizures and symptoms related to behavior.[3,4] These syndromes are rare and the pathogeneses are still not clearly elucidated.[5] In some cases, an association of SW with KTW syndrome seems to exist or there is a clinical and biological overlap between the two diseases. The complexity of the disease phenotypes shows that a classification based on eponymous categories does not enable resolution of nosological problems. There is even a suggestion that SW and KTW are the same diseases with different manifestations, but it should be considered as separate entities.

Both diseases occur almost always sporadically, but a dominant autosomal inheritance has already been described in some families.[6–8] Only some cases of KTW result from mutations of the *VG5Q* gene (formally named AGGF1), or from translocations involving this gene. There are suggestions that the majority of cases result from somatic mutations involving genes that play significant roles in embryonic vasculogenesis and angiogenesis.[4,9,10]

## Case Report

**Figure 1:**
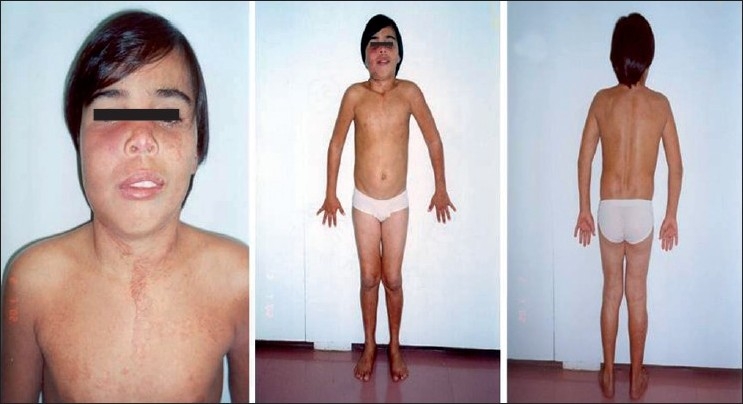
Front and back views of the patient highlighting the hemangioma on the face and hypertrophy.

**Figure 2:**
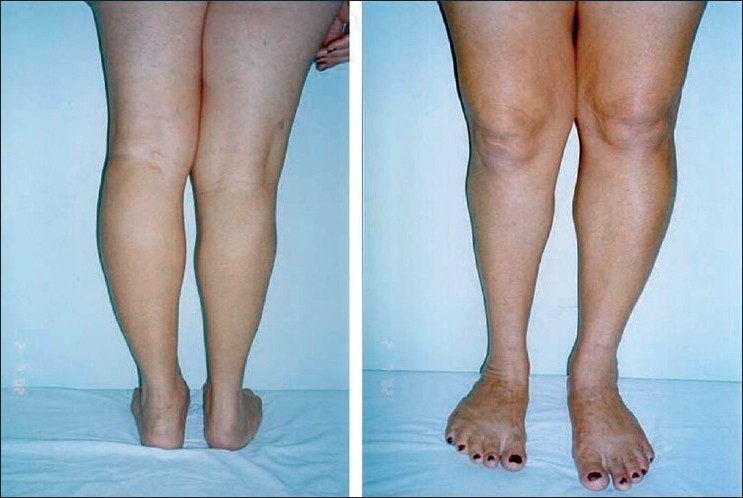
Front and back views of the mother lower limbs with highlighting hypertrophy of the left leg.

This work describes a 16-year-old male patient consulted in the Vascular Surgery and Genetics Departments of the Medical School in São José do Rio Preto, São Paulo, Brazil (FAMERP). The patient presented with a clinical overlap between the SW and KTW syndromes. An interdisciplinary evaluation of the patient and the complementary examinations (angiography, retinography, ultrasonography, radiography, scanometry, electroencephalography and computed tomography) revealed the presence of low stature (<3%), extensive hemangiomas on the face, trunk and limbs, asymmetrical hypertrophy of the cranium, meningeal hemangiomas, glaucoma, enlarged kidney and pyelonephritis of the renal pelvis of the left kidney and varicose veins on the legs [Figure 1]. A genealogical study showed that his mother presented with hypertrophy of the left leg which had not been diagnosed previously [Figure 2]. Both individuals presented with normal karyotypes. Mutations of the *VG5Q* gene were not evaluated.

## Discussion

The present study shows hemihypertrophy of the leg of a woman and the association of Sturge–Weber with Klippel–Trenaunay–Weber syndromes in her son. We question whetherthe anatomical differences of these malformations could be caused by the intensity of genetic transmission of the same genetic alteration. A dominant inheritance pattern is the most satisfactory explanation for the findings in this family. The SW and KTW syndromes appear to be different manifestations of the same affliction. Familial aggregation exists and transmission may be almost imperceptible between generations. Identification of minor manifestations may prove to be a valuable contribution in genetic counseling of families and the prevention of new cases.
